# Incidence, Characteristics, and Prognosis of Incidentally Discovered Hepatocellular Carcinoma after Liver Transplantation

**DOI:** 10.1155/2016/1916387

**Published:** 2016-06-15

**Authors:** Walid El Moghazy, Samy Kashkoush, Glenda Meeberg, Norman Kneteman

**Affiliations:** ^1^Division of Transplantation, Department of Surgery, University of Alberta, Edmonton, AB, Canada T6G 2B7; ^2^Department of Surgery, Sohag University, Sohag 82524, Egypt; ^3^Department of Surgery, National Liver Institute, Menoufia University, Menoufia 32721, Egypt

## Abstract

*Background*. We aimed to assess incidentally discovered hepatocellular carcinoma (iHCC) over time and to compare outcome to preoperatively diagnosed hepatocellular carcinoma (pdHCC) and nontumor liver transplants.* Methods.* We studied adults transplanted with a follow-up of at least one year. Patients were divided into 3 groups according to diagnosis of hepatocellular carcinoma.* Results.* Between 1990 and 2010, 887 adults were transplanted. Among them, 121 patients (13.6%) had pdHCC and 32 patients (3.6%) had iHCC; frequency of iHCC decreased markedly over years, in parallel with significant increase in pdHCC. Between 1990 and 1995, 120 patients had liver transplants, 4 (3.3%) of them had iHCC, and only 3 (2.5%) had pdHCC, while in the last 5 years, 263 patients were transplanted, 7 (0.03%) of them had iHCC, and 66 (25.1%) had pdHCC (*P* < 0.001). There was no significant difference between groups regarding patient survival; 5-year survival was 74%, 75.5%, and 77.3% in iHCC, pdHCC, and non-HCC groups, respectively (*P* = 0.702). Patients with iHCC had no recurrences after transplant, while pdHCC patients experienced 17 recurrences (15.3%) (*P* = 0.016).* Conclusions.* iHCC has significantly decreased despite steady increase in number of transplants for hepatocellular carcinoma. Patients with iHCC had excellent outcomes with no tumor recurrence and survival comparable to pdHCC.

## 1. Introduction

Incidental hepatocellular carcinoma (iHCC) is defined as preoperatively undiagnosed hepatocellular carcinoma (HCC) found on explant pathology of patients transplanted for benign disease. It is not uncommon [1].

Recent advancements in diagnostic imaging have resulted in a marked increase in sensitivity for detection of smaller lesions [2], which should logically be reflected in the incidence of iHCC. So, we aimed in this study to assess iHCC over different time periods, to compare outcomes to preoperatively diagnosed HCC (pdHCC) and to nontumor patients, and to highlight the potential future change in the selection criteria for liver transplantation in view of the continuous improvement in diagnostic imaging.

## 2. Patients and Methods

This study included all adult patients who underwent liver transplantation at University of Alberta Hospital between October 1990 and December 2010 and had at least one year of follow-up. Thirty-two patients had incidentally diagnosed HCC (iHCC). We divided patients into three groups: patients with iHCC presented group 1, group 2 included recipients with pdHCC, and group 3 presented patients transplanted due to non-HCC indications. We compared the groups regarding incidence, characteristics of patients, histopathological characteristics, recurrence, and patient survival. Data were collected prospectively in an electronic database (OTTR, Hickman Kenyon Systems, Omaha, NE) and were analyzed retrospectively. We also divided transplants into 5-year periods to study trend of iHCC and pdHCC over time.

### 2.1. Diagnosis of Hepatocellular Carcinoma

From program inception in 1989, screening of cirrhotic patients on the waiting list was done on an irregular basis and then was done using abdominal ultrasound and serum alpha-fetoprotein (AFP). Since 2006, screening has been done using contrast-enhanced computed tomography or magnetic resonance imaging: every 6 months for cirrhotic patients on the waiting list with no known HCC and every 3 months for patients who have known HCC. Preoperative diagnosis of HCC depended on contrast-enhanced computed tomography and/or magnetic resonance imaging, with biopsy only in cases with nondiagnostic imaging. A lesion was considered HCC if it showed enhancement in the early arterial phase with washout in the late phase or if the lesion had marked increase in size with follow-up, according to AASLD guidelines [1]. A small stable nonenhancing lesion was not considered HCC. TTV was calculated as the sum of the volumes of each tumor (4/3 ×  *πr* ^3^) based on the maximum diameter of each tumor [2].

### 2.2. Statistical Analysis

Data were summarized as mean ± SD for continuous variables and frequency (%) for categorical factors. Groups were compared using one-way ANOVA and chi-square test. Post hoc test (Bonferroni method) was used for paired comparisons. Survival curves were done using the Kaplan-Meier method and compared using the Log-rank test. *P* value < 0.05 was considered significant and all statistics were done using SPSS (SPSS Inc., US) version 16.

## 3. Results

This cohort includes 887 adult patients (>18 years old) who were transplanted at our institution. A total of 734 (82.8%) patients were transplanted due to non-HCC indications and 153 (17.2%) patients had HCC; of them 121 (13.6%) patients were pdHCC and 32 (3.6%) had been diagnosed based on explant pathology (iHCC). Patients with iHCC represented 21% of all HCC cases. Mean age at time of transplant was 50.9 ± 10.6 years with a follow-up of 4.5 ± 4.1 years.

**Figure 1:**
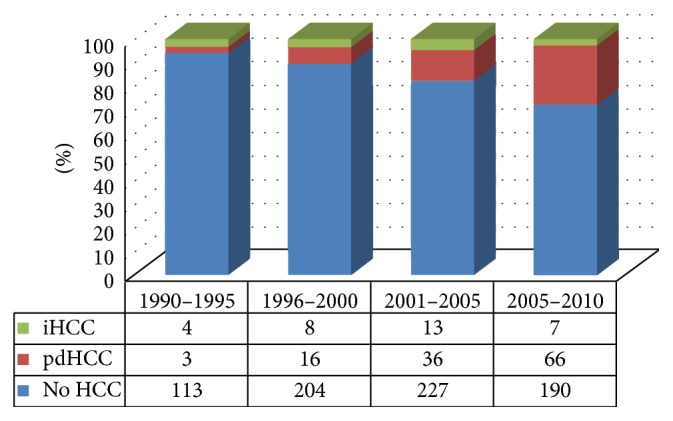


We divided patients into 5-year cohorts and found a steady increase in the number of patients transplanted due to pdHCC. The relative frequency of iHCC has decreased markedly in recent years. Between 1990 and 1995, a total of 120 patients had liver transplants, 4 (3.3%) of them had iHCC, and only 3 (2.5%) had pdHCC. These figures changed dramatically between 2006 and 2010; 263 patients underwent transplant, 7 (0.03%) of them had iHCC, and 66 (25.1%) had pdHCC (*P* < 0.001) (Figure 1).

### 3.1. Comparison of Patient Characteristics in the Three Groups

Comparing the three groups, patients who had been transplanted due to non-HCC indications were significantly younger than patients who had pdHCC and iHCC at time of transplant. Interestingly, patients with iHCC and pdHCC had comparable ages at time of transplant. Males represented 84% and 88% of iHCC and pdHCC groups, respectively, while constituting 58% of non-HCC patients (<0.001). Patients with iHCC and pdHCC had significantly higher HCV infection (<0.001). HBV infection was significantly more prevalent among pdHCC patients compared to patients with iHCC and non-HCC patients (*P* < 0.001). Autoimmune liver diseases including primary sclerosing cholangitis, primary biliary cirrhosis, and autoimmune hepatitis were significantly higher in nontumor patients (<0.001). Prevalence of alcoholic liver disease was comparable in the 3 groups (*P* = 0.109). Patients who had pdHCC had significantly better condition at time of transplant as reflected by the lower MELD and Child-Pugh scores (Table 1).

We compared iHCC and pdHCC groups regarding tumor characteristics and found that patients in the former group had lower alpha-fetoprotein levels, smaller tumor diameters, and smaller total tumor volume as expected (Table 1).

### 3.2. Patient Survival and Tumor Recurrence

**Figure 2:**
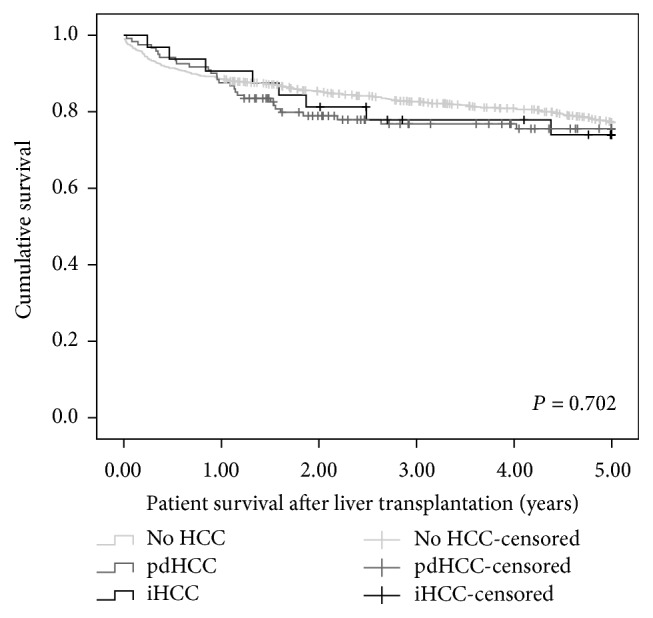


There was no significant difference between groups regarding patient survival, with 1-year survival and 5-year survival in iHCC patients being 87.5% and 74%, respectively, and in pdHCC patients they were 86.8% and 75.5%, respectively, and in patients transplanted for non-HCC indications they were 88.5% and 77.3% (*P* = 0.702) (Figure 2).

Patients with iHCC had 12 (37.5%) cumulative deaths with a mean time from transplant to death of 3.5 ± 3.1 years. The cause of death was bacterial infection in 3 patients, malignancy in 2 patients (metastatic pancreas cancer and posttransplant lymphoproliferative disease), chronic rejection in two patients, multiorgan failure in 2 patients, cardiovascular and neurological complications in 2 patients, and suicide in one.

In pdHCC, 33 patients (27.3%) died, with a mean time from transplant to death of 3.0 ± 3.9 years. Causes of death included recurrence of HCC in 14 patients, other malignancies in 2 patients, cardiovascular disease in 5 patients, and bacterial infections in 3 patients. The remaining patients were lost due to hemorrhage (1 patient), recurrence of primary disease (2), chronic rejection (1), multiorgan failure (2), neurological complications (1), and pulmonary complications (2). In the non-HCC patients, 256 died during the follow-up period.

**Figure 3:**
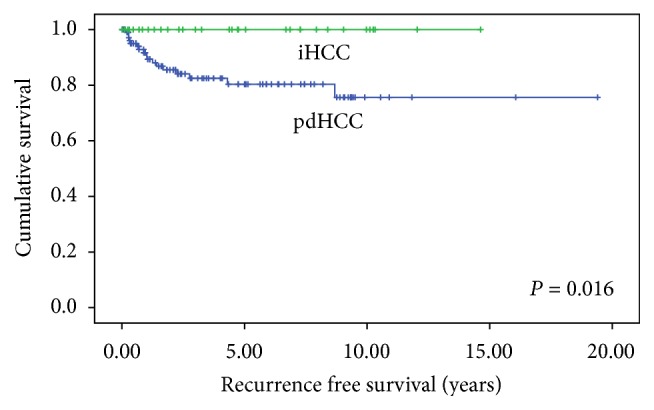


Patients with iHCC had no tumor recurrences during the follow-up, while patients with pdHCC had 17 recurrences (15.3%). Recurrence free survival at 5 years after transplant was 100% in group 1 and 80.4% in group 2 (*P* = 0.016) (Figure 3).

### 3.3. Description of the Incidental HCC Cases

Table 2 summarizes iHCC cases. iHCC lesions varied from 1 to 7 in number; single lesion was the most frequent case: 20 patients (62.5%). The maximal diameter of lesions ranged from 0.6 to 5 cm; 50% of lesions were smaller than 1.5 cm. Total tumor volume ranged from 0.1 to 66.3 cc^3^ with 50% of patients having tumor volume of less than 2.6 cc^3^. Follow-up was done by transplant centers for 17 patients and nontransplant centers for 15 patients. Initially, abdominal ultrasound and serum AFP were used for screening; the highest pretransplant AFP ranged from 3 to 41. Since 2006, screening for all patients on waiting list is done using contrast-enhanced CT or magnetic resonance imaging (MRI) scans every 6 months with AFP.

## 4. Discussion

In this study, we analyzed the outcome of 887 adult patients who received liver transplant in our center over a period of 20 years and found that 32 out of the 153 patients with HCC were diagnosed by pathological examination of the explant only. No patient with iHCC experienced recurrence of malignancy and survival was comparable to patients with pdHCC, perhaps because of more severe preoperative liver disease in the iHCC group as reflected by higher MELD and Child-Pugh scores.

Over a period of 20 years, incidence of HCC has increased steadily. In the first 5 years, only 2.5% of transplant patients had pdHCC, while in the last five years, 25% of cases had pdHCC. Despite increasing incidence of HCC over time, incidentally discovered HCC has decreased significantly. In the first five years, iHCC represented 3.3% of cases, while in the last five years iHCC dropped to 0.03%. iHCC represented 21% of all cases diagnosed with HCC and constituted 3.6% of all adult transplanted patients which is comparable to the published literature, where prevalence of iHCC among transplanted HCC cases has varied from 4.2% to 40% [3–6]. This reflects the challenge in diagnosis of HCC in the presence of underlying liver cirrhosis, especially for lesions smaller than 2 cm. It is difficult to differentiate small HCC from dysplastic nodules, which results in discrepancy between the pretransplant imaging and the histopathologic examination of explanted livers [7]. Kishi et al. [6] found that 40% of HCCs were either missed or underestimated by the pretransplant imaging.

In our cohort, the relative proportion of iHCC among all transplanted patients with HCC decreased steadily from 62.5% in the first 5 years to reach 14.7% in the last 5 years. This dramatic decline likely reflects both significant improvement in diagnostic imaging and increasing experience in the diagnostic teams as well as close monitoring of patients. Reported sensitivities of contrast-enhanced MRI for detection of HCC of all sizes are 33%–90% and 50%–80% for lesions smaller than 2 cm and 4%–33% for lesions smaller than 1 cm [8, 9]. These results represent improved imaging from an older report that showed sensitivity of 21% and 0% for detection of lesions of 1-2 cm and <1 cm, respectively. Sensitivity for detection of small HCC had further improvement with use of double-contrast MR imaging that has been shown to detect HCC with sensitivities of 92% and 38% for lesions measuring 1-2 cm and less than 1 cm, respectively [10].

However, not all lesions were small; some of the missed lesions were larger than 2 cm, which should raise another issue about periodic screening for patients at risk of developing HCC and also about where to do the screening. Since 2006, our center started to do screening for cirrhotic patients on the liver transplant waiting list every 6 months using contrast-enhanced CT or MR scans with serum AFP which was associated with an increased incidence of pdHCC in parallel with decreasing incidence of iHCC. Few cases that followed the protocol had small lesions that had enhancement with contrast but were atypical in appearance and difficult to characterize. One patient had a large iHCC that was missed on imaging; this patient had screening by his primary care center. Reviewing iHCC cases revealed that about half of them were screened by primary care centers. A study from Japan [11] showed that doing the surveillance at a specialized liver center resulted in a higher chance of identifying HCC at an early stage.

In this study, we did not find dramatic difference regarding incidence of iHCC among different liver diseases. In East Asian countries, there is high prevalence for HCC among HBV carriers. A study from Hong Kong [12] showed a higher incidence of iHCC among patients with hepatitis B as 10% of the population are carriers for hepatitis B, and HBV-related cirrhosis represents the main indication in 90% of transplanted cases in that center. In our cohort, iHCC was significantly higher among HCV patients and lower between HBV and autoimmune liver diseases. pdHCC had a significantly higher incidence among HBV patients compared to iHCC and non-HCC patients, perhaps due to a more effective screening program in the general medical community. High-risk patients clearly require close follow-up for early detection of HCC; screening includes tumor markers (AFP) and ultrasound in most centers. AFP has a low sensitivity for detecting HCC; its clinical utility has also been eroded somewhat by recent improvement of imaging [13]. Using two or more tumor markers AFP, protein induced by Vitamin K absence or antagonist-II (PIVKA-II), or AFP lectin fraction (AFP-L3) has been associated with increased sensitivity for detection of HCC [14].

Outcome of iHCC varies between centers. In a study of 198 HCC patients [3], 51 patients had iHCC, and the authors did not find a survival advantage for iHCC patients (*P* = 0.084). Patient survival at 1 year and 5 years was 78% and 58% in iHCC group and 90% and 70% in pdHCC group. They had recurrence in 13% of iHCC patients and 22% recurrence in pdHCC patients (*P* = 0.486). In another study of 53 patients [15], 11 patients had iHCC and they had better survival than the 42 patients who had pdHCC (*P* < 0.05). Survival at 1 year and 5 years was 82% and 82% in iHCC group and 78% and 57% in pdHCC patients. In our cohort, iHCC patients did not have any recurrence during the follow-up; however, they did not have a survival advantage over patients with pdHCC. Survival at 1 year and 5 years was 90.6% and 72.5% in iHCC group and 86.3% and 71.9% in pdHCC group, respectively. We suspect that the iHCC patients did not achieve better survival than pdHCC patients due to their poorer general condition at time of transplant as reflected from their significantly higher MELD and Child-Pugh scores, notwithstanding the reality that high MELD score is a weak prognostic factor for mortality of HCC patients after liver transplantation [16]. Patients with pdHCC receive MELD exception points and hence are transplanted with relatively preserved liver function and better general condition than patients with a non-HCC primary listing diagnosis.

It could be expected that most of all incidental HCC would be within Milan criteria. Surprisingly, we found 24% of our iHCC cohort was beyond Milan based on the explant pathology. Six out of 8 cases were beyond Milan based on the number of lesions. All iHCC cases were within the TTV/AFP criteria utilized in our center since 2007, which are based on total tumor volume of <115 cc^3^ and alpha-fetoprotein of <400 ng/mL [17]. As the ability of diagnostic imaging to detect early HCC continues to improve, the term “incidental HCC” may become a historical one in the future. Patients with multiple small HCC can have excellent outcome in terms of recurrence and patient survival as shown in this study and previously published reports from other centers [3, 6, 15, 17]. This study provides additional support for not excluding HCC patients from transplant based on the number of lesions given the increasing sensitivity of imaging over time.

In conclusion, we found patients with iHCC to have excellent tumor related outcome with no tumor recurrence and survival comparable to non-HCC patients and pdHCC patients. The incidence of iHCC has decreased dramatically over years, while the incidence of HCC as the primary listing diagnosis for liver transplantation has continued to increase in many centers. This study provides further evidence that the number of HCC lesions should not be used alone for excluding patients from transplantation, as it may exclude patients with favorable outcome.

## Figures and Tables

**Table 1:** Characteristics of patients.

Variables	iHCC (*n* = 32)	pdHCC (*n* = 121)	Nontumor (*n* = 734)	*P* value
Age	54.0 ± 7.1	56.2 ± 7.0	49.9 ± 10.9	<0.001

Gender (female : male), *n* (%)	5 : 27 (16 : 84)	15 : 106 (12 : 88)	305 : 429 (42 : 58)	<0.001

Underlying liver disease				
HCV (yes/no), *n* (%)	17 : 15 (53 : 47)	67 : 54 (55 : 45)	204 : 530 (28 : 72)	<0.001
HBV (yes/no), *n* (%)	2 : 30 (6 : 94)	28 : 93 (23 : 77)	34 : 700 (5 : 95)	<0.001
Alcoholic liver disease (yes/no), *n* (%)	14 : 17 (44 : 56)	37 : 84 (31 : 69)	216 : 518 (29 : 71)	0.109
Autoimmune liver diseases (yes/no), *n* (%)	3 : 29 (9 : 91)	4 : 117 (3 : 97)	219 : 515 (30 : 70)	<0.001

MELD at time of transplant	20.1 ± 6.7	14.6 ± 7.4	20.5 ± 10.8	0.001

Child-Pugh score	10.9 ± 2.2	8.5 ± 2.6	9.96 ± 2.3	<0.001

Waiting time (days)	121.4 ± 152.2	203.1 ± 284.2	191 ± 176	0.039

Number of lesions	1.9 ± 1.6	2.8 ± 3.8	—	0.058

Maximum diameter	2.0 ± 1.3	3.5 ± 1.9	—	<0.001

Milan (within : beyond)	25 : 8 (78 : 22)	59 : 52 (49 : 51)	—	0.021

Total tumor volume (explant)	12.2 ± 19.1	47.8 ± 108.6	—	0.002

Lowest AFP before transplant	7.7 ± 6.9	98.5 ± 381.7	—	0.025
Median (range)	4 (1–28)	12 (2–3289)		

Highest AFP before transplant	14.2 ± 10.3	378.8 ± 1103.2	—	0.002
Median (range)	11 (3–41)	37 (4–8987)		

Vascular invasion (no/yes), *n* (%)	25 : 8 (78 : 22)	67 : 37 (55 : 45)	—	0.227

**Table 2:** Characteristics of iHCC cases.

Case number	Transplant year	Diagnosis	Number of lesions	Diameter of largest nodule (cm)	TTV	Highest AFP	Method of screening	Screening center
1	1994	AC	1	4.0	35.5	—	US	Tertiary
2	1995	HCV, AC	1	1.5	1.8	—	US	Primary
3	1995	HCV, Hemo.	1	0.8	0.3	—	US	Primary
4	1995	HCV	1	0.8	0.3	10	US	Tertiary
5	1998	HBV, AC	4	3.8	33.4	4	US	Tertiary
6	1998	AC	3	2.0	6.5	1	CT	Primary
7	1999	HCV	1	0.8	0.3	11	CT	Tertiary
8	1999	PSC	2	5.0	66.3	4	CT	Tertiary
9	1999	HCV	1	4.0	33.5	2	CT	Tertiary
10	2000	Crypt.	1	0.8	0.3	3	CT	Tertiary
11	2000	AC	1	1.0	0.5	3	CT	Tertiary
12	2000	A1AD, HCV	4	4.0	38.7	3	CT	Primary
13	2001	AC	5	3.2	27.5	2	CT	Primary
14	2001	HCV	1	1.5	1.8	30	CT	Tertiary
15	2001	HCV, AC	7	1.5	4.3	2	CT	Tertiary
16	2002	Hemo., AC	5	2.0	4.4	17	CT	Tertiary
17	2002	HCV	1	0.9	0.4	21.1	CT	Tertiary
18	2003	HCV	2	1.1	0.9	16	CT	Primary
19	2003	HCV	2	2.0	9.0	10	CT	Primary
20	2003	HCV, AC	1	2.4	7.2	11	CT	Primary
21	2004	Crypt.	1	1.9	3.6	4	CT	Primary
22	2005	HCV, AC	1	0.7	0.1	41	MRI	Primary
23	2005	HCV, AC	1	0.6	0.1	8	MRI	Tertiary
24	2005	HCV	2	1.7	3.0	15	MRI	Tertiary
25	2005	PBC, AC	5	3.2	33.0	16	MRI	Primary
26	2007	HCV	1	2.3	1.0	4	MRI	Tertiary
27	2007	AC	1	1.5	0.3	3	MRI	Primary
28	2007	Hemo.	1	1.5	2.3	3	MRI	Tertiary
29	2009	PSC	1	0.6	0.1	10	MRI	Primary
30	2009	HBV	2	4.5	61.9	10	MRI	Primary
31	2009	Hemo., NAFLD	1	1.2	0.9	23	MRI	Tertiary
32	2009	HCV, AC	1	1.5	1.7	3	MRI	Primary

AC, alcoholic cirrhosis; PSC, primary sclerosing cholangitis; Hemo., hemochromatosis; Crypt., cryptogenic cirrhosis; A1AD, alpha-1 antitrypsin deficiency; PBC, primary biliary cirrhosis; NAFLD, nonalcoholic fatty liver disease; US, abdominal ultrasound; CT, computed tomography; MRI, magnetic resonance imaging.

## References

[1] Bruix J., Sherman M. (2005). Management of hepatocellular carcinoma. *Hepatology*.

[2] Toso C., Trotter J., Wei A. (2008). Total tumor volume predicts risk of recurrence following liver transplantation in patients with hepatocellular carcinoma. *Liver Transplantation*.

[3] Castillo E., Pelletier S., Kumer S., Abouljoud M., Divine G., Moonka D. (2009). Incidental hepatocellular carcinoma after liver transplantation: population characteristics and outcomes. *Transplantation Proceedings*.

[4] Raphe R., Felício H. C. C., Rocha M. F. (2010). Histopathologic characteristics of incidental hepatocellular carcinoma after liver transplantation. *Transplantation Proceedings*.

[5] Klintmalm G. B. (1998). Liver transplantation for hepatocellular carcinoma: a registry report of the impact of tumor characteristics on outcome. *Annals of Surgery*.

[6] Kishi Y., Sugawara Y., Tamura S., Kaneko J., Kokudo N., Makuuchi M. (2006). Impact of incidentally found hepatocellular carcinoma on the outcome of living donor liver transplantation. *Transplant International*.

[7] Taouli B., Krinsky G. A. (2006). Diagnostic imaging of hepatocellular carcinoma in patients with cirrhosis before liver transplantation. *Liver Transplantation*.

[8] Bhattacharjya S., Bhattacharjya T., Quaglia A. (2004). Liver transplantation in cirrhotic patients with small hepatocellular carcinoma: an analysis of pre-operative imaging, explant histology and prognostic histologic indicators. *Digestive Surgery*.

[9] Willatt J. M., Hussain H. K., Adusumilli S., Marrero J. A. (2008). MR imaging of hepatocellular carcinoma in the cirrhotic liver: challenges and controversies. *Radiology*.

[10] Bhartia B., Ward J., Guthrie J. A., Robinson P. J. (2003). Hepatocellular carcinoma in cirrhotic livers: double-contrast thin-section MR imaging with pathologic correlation of explanted tissue. *American Journal of Roentgenology*.

[11] Ando E., Kuromatsu R., Tanaka M. (2006). Surveillance program for early detection of hepatocellular carcinoma in Japan: results of specialized department of liver disease. *Journal of Clinical Gastroenterology*.

[12] Chui A. K. K., Wong J., Rao A. R. N. (2003). High incidence of incidental hepatocellular carcinoma exists among hepatitic explanted livers. *Transplantation Proceedings*.

[13] Snowberger N., Chinnakotla S., Lepe R. M. (2007). Alpha fetoprotein, ultrasound, computerized tomography and magnetic resonance imaging for detection of hepatocellular carcinoma in patients with advanced cirrhosis. *Alimentary Pharmacology and Therapeutics*.

[14] Kudo M. (2008). Hepatocellular carcinoma 2009 and beyond: from the surveillance to molecular targeted therapy. *Oncology*.

[15] Fernández J. A., Robles R., Marin C. (2003). Can we expand the indications for liver transplantation among hepatocellular carcinoma patients with increased tumor size?. *Transplantation Proceedings*.

[16] Ioannou G. N., Perkins J. D., Carithers R. L. (2008). Liver transplantation for hepatocellular carcinoma: impact of the MELD allocation system and predictors of survival. *Gastroenterology*.

[17] Toso C., Asthana S., Bigam D. L., Shapiro A. M. J., Kneteman N. M. (2009). Reassessing selection criteria prior to liver transplantation for hepatocellular carcinoma utilizing the scientific registry of transplant recipients database. *Hepatology*.

